# The Silent Pandemic: Antimicrobial Resistance, a Global Threat with Ancient Roots

**DOI:** 10.3390/antibiotics15060560

**Published:** 2026-05-31

**Authors:** Davide Orsini, Anna Maria Spagnolo, Mariano Martini

**Affiliations:** 1 Department of Life Sciences, Health, and Health Professions, Link Campus University, 00165 Rome, Italy; 2 Department of Health Sciences, University of Genoa, 16132 Genoa, Italy; am.spagnolo@unige.it (A.M.S.); mariano.yy@gmail.com (M.M.)

**Keywords:** pandemic, antimicrobial resistance, Alexander Fleming, history of medicine, microbes

## Abstract

A silent pandemic is underway that is causing more than a million deaths each year worldwide: antimicrobial resistance. According to World Health Organization forecasts, this phenomenon could become the leading cause of death by 2050. It represents one of the most pressing global health threats, compromising the effectiveness of life-saving therapies and making infections increasingly difficult to treat. Although antimicrobial resistance is often considered a recent phenomenon, it was first recognized in the early stages of antimicrobial use, with reports predating Alexander Fleming’s discovery of penicillin in 1928. In this context, the present review examines selected representative cases of the early recognition of antimicrobial resistance through the analysis of the initial evidence of microbial adaptation associated with the use of pre-antibiotic chemotherapeutic agents such as Salvarsan, as well as the first reports of reduced clinical efficacy of sulfonamides and penicillin. These observations have contributed to the current understanding of resistance as a dynamic process driven by microbial adaptive mechanisms and amplified by the selective pressure exerted by antimicrobial use.

## 1. Introduction

Antimicrobial resistance (AMR) is a pandemic underway that is rather silently causing more than a million deaths worldwide every year, and approximately 35,000 in Europe alone [1,2].

According to a Global Burden of Disease (GBD) study, by 2050 antimicrobial resistance could result in approximately 1.91 million (1.56–2.26) attributable deaths and 8.22 million (6.85–9.65) associated deaths globally [3,4].

It is a growing threat to global health, undermining the effectiveness of life-saving treatments and placing populations at heightened risk, whether from common infections or routine medical interventions [5,6].

Resistant bacterial infections are consequently more difficult to treat [7], while, at the same time, the development pipeline for new antibiotics remains limited [8].

Since 2017, 13 new antibiotics targeting priority bacterial pathogens have received regulatory approval, several of which are now included in the WHO Essential Medicines List. Nevertheless, antimicrobial resistance continues to evolve, with increasingly complex resistance profiles emerging, including diminished susceptibility even to recently introduced agents [9].

In hospital environments, the dissemination of antimicrobial-resistant pathogens can lead to nosocomial outbreaks, especially in intensive care units, where vulnerable patients and invasive procedures facilitate rapid transmission [10,11,12].

Antibiotics are among the most important discoveries of the 20th century, having saved millions of lives, protected populations from numerous infectious diseases, and substantially reduced human suffering.

However, since their discovery, resistance to these drugs has been observed in various microorganisms.

Antibiotic resistance in bacteria can be either intrinsic or acquired. Intrinsic resistance is exhibited due to the intrinsic properties of the bacterium. Examples of this resistance include resistance to glycopeptides exhibited by Gram-negative bacteria due to the impermeability of their outer membrane in the cell envelope. In contrast, acquired resistance occurs when a previously susceptible bacterium acquires a resistance mechanism through mutation or the acquisition of new genetic material via horizontal gene transfer, most commonly through conjugation mediated by plasmids carrying resistance genes. Importantly, the widespread use and misuse of antibiotics have exerted strong selective pressure, driving the emergence and dissemination of acquired antimicrobial resistance among microbial populations [13].

Today, the increasing ability of microorganisms to withstand antibiotic treatment could render currently manageable conditions, such as post-operative infections, infectious diseases and complications of chronic wounds, once again lethal or severely debilitating.

From this perspective, global antimicrobial resistance today appears to be one of the most important public health problems facing both human and veterinary medicine [14,15].

The causes of antimicrobial resistance are multifactorial and arise from the complex interplay between clinical practices, environmental factors, and human behaviours, which collectively contribute to the selection and dissemination of resistant microorganisms [16].

The inappropriate use of antimicrobial agents in humans, animals, and agriculture is one of the main factors accelerating the process.

This complex interaction between multiple causes and actors aggravates the situation, with a significant impact also attributable to pollution and weather conditions, as well as to the conflicts that are ravaging many parts of the world [17]. Indeed, the poor conditions of hospital infrastructures, which are frequently targeted by hostile forces, inappropriate therapies, high levels of heavy metal contamination in the environment and the lack of clean water and sanitation are all contributing to raising antibiotic resistance to unprecedented levels.

It therefore constitutes one of the main health challenges of the coming decades, requiring constant monitoring and coordinated mitigation strategies [13].

In this regard, the Global Antimicrobial Resistance and Use Surveillance System (GLASS), established by the WHO, is monitoring the situation, “to strengthen evidence on AMR through standardized data collection, analysis, sharing and reporting” [5,18].

However, given the complexity and multidimensional nature of antimicrobial resistance, surveillance alone is not sufficient. Effective control requires a coordinated, multi-sectoral approach, as resistant microorganisms and resistance determinants can disseminate across human, animal, and environmental compartments.

Accordingly, in 2015 the WHO promoted the Global Action Plan on antimicrobial resistance [19], recognizing that a problem of such complexity needs to be addressed with coordinated multi-sectoral interventions proper to the “One Health” approach, integrating the human, veterinary, and environmental sectors [20,21,22].

In this context, the “One Health” approach has emerged as a key strategy, promoting integrated interventions such as antimicrobial stewardship, strengthened surveillance systems, improved diagnostics, and the development of innovative therapeutic options to mitigate the global spread of antimicrobial resistance [23,24,25].

It is important to emphasize that antimicrobial resistance is not a recent phenomenon: the earliest observations of resistance to antimicrobial compounds date back to a period prior to Alexander Fleming’s discovery of penicillin in 1928 [26].

In light of this historical evidence, the aim of this review is to examine selected representative cases of the early recognition of antimicrobial resistance, through the analysis of the first evidence emerging after the use of pre-antibiotic chemotherapeutic agents such as Salvarsan, up to the earliest documented failures of sulfonamides and penicillin.

Through the analysis of these key milestones, this review highlights how microbial adaptation to antimicrobial compounds has been observed since the very early stages of antimicrobial chemotherapy. These observations have contributed to the current understanding of antimicrobial resistance as a dynamic process driven by microbial adaptive mechanisms and amplified by the selective pressure exerted by antimicrobial use.

## 2. Early Observations of Antimicrobial Resistance: The Case of “Salvarsan”

In 1924, the first clinical case of resistance to Salvarsan was recorded in a patient with syphilis. Salvarsan (or arsphenamine or compound 606) is a drug derived from arsenic, and acts against syphilis and African trypanosomiasis. This drug was the fruit of intense research carried out by the German bacteriologist Paul Ehrlich (1854–1915), who won the Nobel Prize in 1908 together with the Russian immunologist Il’ja Il’ič Mečnikov (1845–1916) for their research in immunology [27].

Salvarsan was the first synthetic antibacterial chemotherapy drug, which was effective against *Treponema pallidum*, the causative agent of syphilis: immediately, it had great success in the treatment of syphilis, being far more efficacious than traditional mercurial therapies [28]. Ehrlich went on to test further compounds and improved Salvarsan; the newer version, “compound 914” or Neosalvarsan, was more soluble, had a lower arsenic content, and proved less toxic and more active. It came onto the market in 1913 and proved to be the most efficacious drug for the treatment of syphilis until the advent of antibiotics.

Between 1907 and 1909, Paul Ehrlich himself observed in the laboratory that *Trypanosoma*, the parasite that causes sleeping sickness, developed resistance after repeated exposure to sub-lethal doses of arsenic compounds, the progenitors of Salvarsan.

“He showed that resistance, once acquired, was stably inherited and in 1908 proposed that resistance was due to ‘reduced avidity of the chemoreceptors so that they are no longer able to take up’ the drug” [29,30].

The same phenomenon was observed on treatment with Salvarsan; owing to the prolonged, and often interrupted, use of the drug (the therapy lasted about 18 months), *Treponema pallidum* developed the ability to survive, via a mechanism similar to the one that would later be demonstrated.

The Salvarsan experience was therefore an early example of antibiotic resistance and demonstrated that the ability of microorganisms to adapt to drugs was a natural phenomenon, which was known long before the advent of antibiotics.

About 10 years later, in 1919, the researcher S.M. Neuschlosz made one of the first significant experimental contributions to understanding drug inactivation when he demonstrated that *Paramecium caudatum* was able to develop resistance to quinine and other compounds through a mechanism of enzymatic inactivation (detoxification), and not simply through physiological adaptation. “Neuschlosz reported that Paramecium caudatum resistant to quinine and to certain dyes acquired the ability to destroy the toxic agents” [31].

His study constituted an important step forward in our understanding of drug metabolism by demonstrating that organisms can inactivate foreign substances through biotransformation: specific enzymes metabolize the compounds, reducing or eliminating their therapeutic effect.

## 3. Resistance to Sulfonamides

In the period between Alexander Fleming’s discovery (1928) and the possibility of using penicillin (mid-1940s), an equally interesting study was undertaken, which led to the introduction of sulfonamides into therapy. Unlike antibiotics of natural origin, these antibacterial chemotherapeutic drugs are obtained by means of chemical synthesis.

Towards the end of the 1920s, the German company Bayer, which in 1925 had merged with other German chemical companies to form IG Farben, launched a programme of research in antibacterial chemotherapy, in the wake of the success of Salvarsan. Specifically, Bayer researchers highlighted the antimicrobial activity of certain azo dyes that had previously been used in the textile industry. In December 1932, they filed a patent application for *Sulfamidochrysoidine*.

Synthesized by the chemists Josef Klarer (1898–1953) and Fritz Mietzsch (1896–1958), this molecule was passed on to Gerhard Domagk (1895–1964), a German chemist and the research director at Bayer’s Laboratory of Experimental Pathology and Bacteriology.

Domagk demonstrated that this substance had antibacterial properties, that is, it was capable of destroying streptococci. He announced his findings on 15 February 1935, and published the results of his studies on what was henceforth referred to as Prontosil rubrum [32,33].

On the basis of Domagk’s discovery, a group of researchers from the Pasteur Institute, including Jacques Tréfouël (1897–1977), Daniel Bovet (1907–1992) and Federico Nitti (1905–1947), demonstrated that Prontosil was metabolised in the body, through a process of “bioactivation”, undergoing metabolic degradation in the liver, which led to the synthesis of the actual antibacterial molecule, i.e., the synthesis of sulfanilamide.

**Figure 1:**
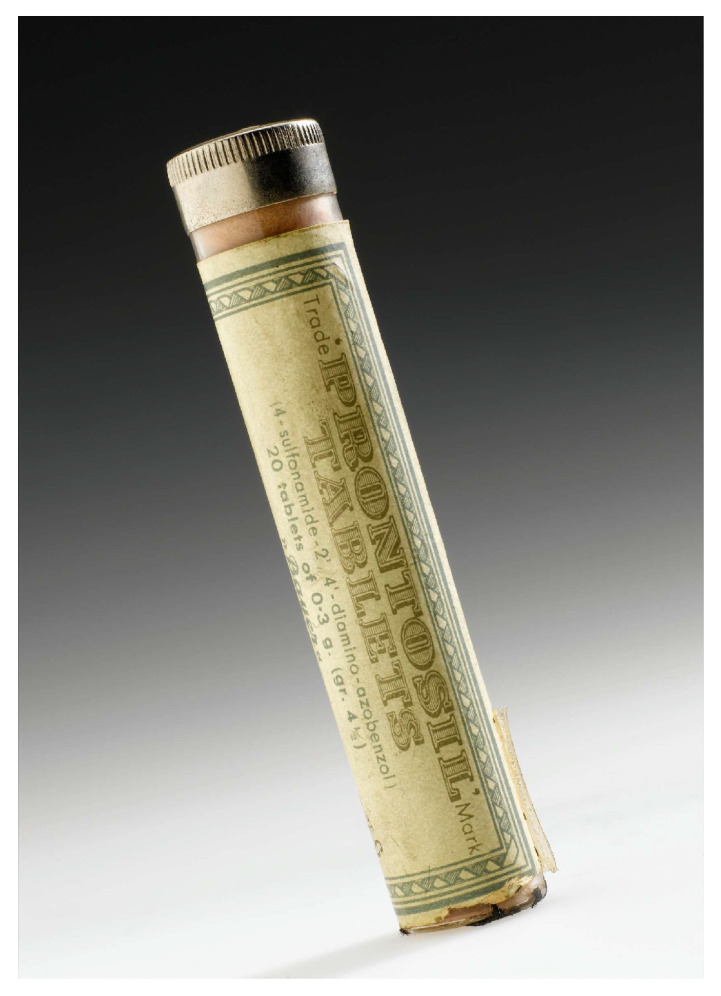
Tube of Prontosil tablets available in Germany during 1935–1950 (Public domain).

It was thus understood that Prontosil was actually a precursor of the effective drug. Since the antibacterial properties were not linked to the characteristics of the product, Bayer directly marketed sulfanilamide under the name Prontosil album (Figure 1), which was used to treat bacterial infections, including puerperal sepsis, before the advent of modern antibiotics such as penicillin [34].

The introduction of sulfonamides constituted a true revolution in therapy, transforming medicine from the 1930s onward [35]. Pneumonia, sepsis and puerperal infections could finally be treated with these drugs. In the following years, new sulphonamides were synthesized which were increasingly active and less toxic, such as sulfapyridine, which was used to treat Winston Churchill’s pneumonia in 1943 [36] and sulfathioazole, which served as the parameter for evaluating the antimicrobial activity of all new anti-infective agents for over two decades. This led to the massive use of sulphonamides, sometimes prophylactically, especially on the battlefields of the Second World War: such a widespread use that resistant microorganisms appeared within a few years.

Bacterial resistance had, however, already been observed in 1937 in cases of gonorrhea treated with sulfonamide drugs at Johns Hopkins Hospital in Baltimore, USA [37,38]. By 1944, this resistance had increased significantly, with treatment failure rates exceeding 30%. The doctors who reported this resistance were so concerned by the rapidity with which the phenomenon had manifested itself that they spoke out against the indiscriminate use of the drug, which, they asserted, should be only available on medical prescription. Despite this, by the late 1940s, over 90% of samples of the bacterium *Neisseria gonorrhoeae* taken from infected patients and cultivated in the laboratory were found to be resistant to sulfonamides in vitro. Moreover, around 1938–1939, reports came in of strains of *Streptococcus* that no longer responded to the bactericidal effects of Prontosil [39]. Because of these cases of resistant strains and the availability of penicillin from the mid-1940s, sulfonamides were rapidly replaced by penicillin, especially in the treatment of penetrating wounds.

The experience of Prontosil, and of sulphonamides in general, is a fundamental page in the history of medicine. Indeed, Prontosil demonstrated that bacterial infections could be treated and prevented, morbidity significantly reduced and mortality rates due to infection reduced. At the same time, however, it quickly revealed the limits of its therapeutic action, owing to the ability of bacteria to adapt.

## 4. The First Forms of Resistance to Penicillin and Alexander Fleming’s Warning

Thanks to the fundamental contributions of Ernst Boris Chain (1906–1979) and Howard Florey (1898–1968), Alexander Fleming’s (1881–1955) discovery of penicillin could be used in clinical therapy.

Chain shared the 1945 Nobel Prize for Physiology/Medicine [40] with Alexander Fleming and Howard Florey [41] “for the discovery of penicillin and its curative effect in various infectious diseases” [42].

Although penicillin first became available for medicinal purposes in the USA in 1943, a strain of *Escherichia coli* [43] that could produce an enzyme (penicillinase) capable of inactivating penicillin had already been identified in 1940 by the biochemists Edward Abraham (1913–1999) and Ernst Boris Chain [44].

This inactivating effect was demonstrated by the following experiment: “A solution of 1 mg. penicillin in 0·8 c.c. of water was incubated with 0·2 c.c. of centrifuged and dialysed bacterial extract at 37° for 3 h, in the presence of ether, and a control solution of penicillin of equal concentration was incubated without enzyme for the same time. […] The penicillin solution incubated with the enzyme had entirely lost its growth-inhibiting activity, whereas the control solution had retained its full strength” [45,46].

**Figure 2:**
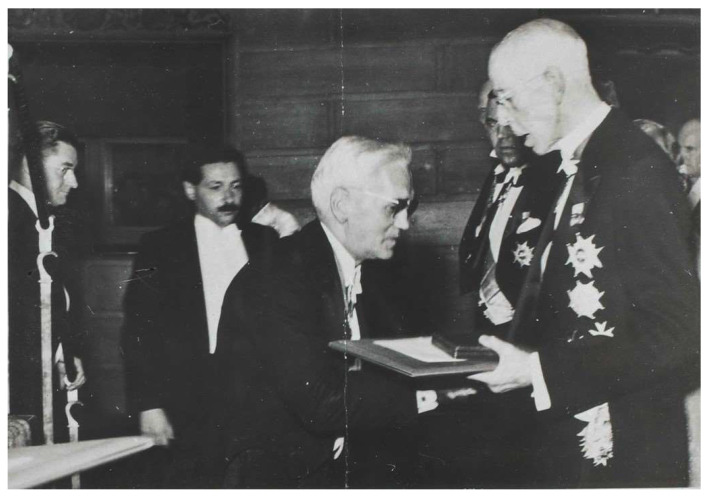
Sir Alexander Fleming receives the Nobel Prize from King Gustaf V of Sweden on 10 December 1945. Behind Alexander Fleming stands Nobel Laureate Ernst B. (Public domain—Wikimedia Commons, Photographer unknown).

A few months later, in 1942, Charles Henry Rammelkamp Jr. (1911–1981) and Thelma Maxon, two researchers at Boston University, documented the resistance of some strains of *Staphylococcus aureus* in hospitalized patients [47]. Through laboratory experiments, they also demonstrated that *Staphylococcus aureus* bacteria could develop resistance to penicillin if successive generations were exposed to weak dilutions of the drug, which meant that increasingly higher doses were required in order to destroy them. Thus, when penicillin was first used in clinical practice, the possibility that bacterial resistance might rapidly develop was already known. Fleming was extremely clear about the possible consequences of this in his speech before the Nobel Prize Committee: he evoked the real possibility that microorganisms could develop resistance to penicillin. Indeed, in the speech he made upon receiving the Nobel Prize in 1945 (Figure 2), Fleming declared:

“But I would like to sound one note of warning. Penicillin is to all intents and purposes non-poisonous so there is no need to worry about giving an overdose and poisoning the patient. There may be a danger, though, in under-dosage. It is not difficult to make microbes resistant to penicillin in the laboratory by exposing them to concentrations not sufficient to kill them, and the same thing has occasionally happened in the body. The time may come when penicillin can be bought by anyone in the shops. Then there is the danger that the ignorant man may easily under-dose himself and, by exposing his microbes to non-lethal quantities of the drug, make them resistant.” [48].

## 5. Mary Barber, the Pioneer in the Fight Against Antibiotic Resistance

**Figure 3:**
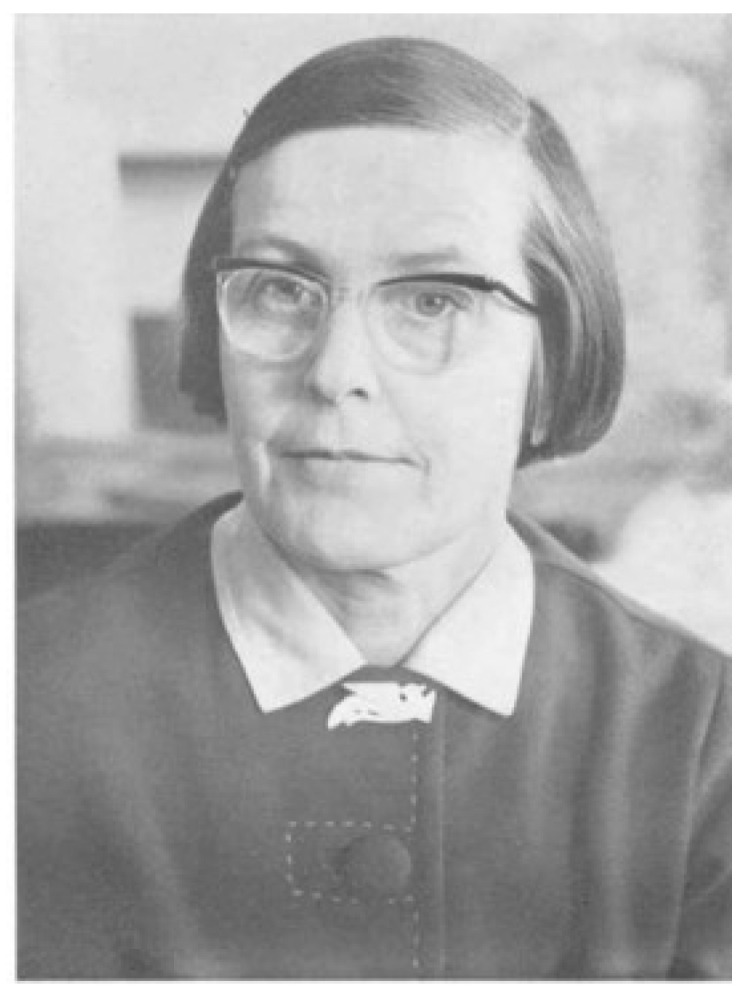
Mary Barber, a pioneer who documented the phenomenon of penicillin resistance early on (Public domain).

Before long, Fleming’s predictions came true. Indeed, between 1946 and 1948 at London’s Hammersmith Hospital, the English bacteriologist Mary Barber (1911–1965) (Figure 3) observed strains of *Staphylococcus pyogenes* that were resistant to penicillin. Barber was one of the pioneers in this field, documenting the phenomenon of penicillin resistance early on shortly after the drug was introduced into the hospital.

**Figure 4:**
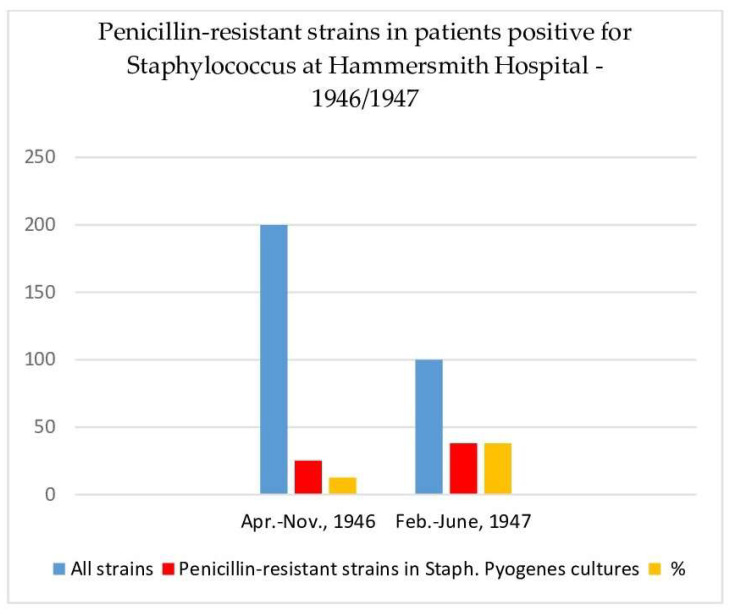
Penicillin-resistant strains in patients positive for Staphylococcus at Hammersmith Hospital—1946/1947 (Source: Mary Barber, *Staphylococcal infection due to Penicillin-resistant strains*).

**Figure 5:**
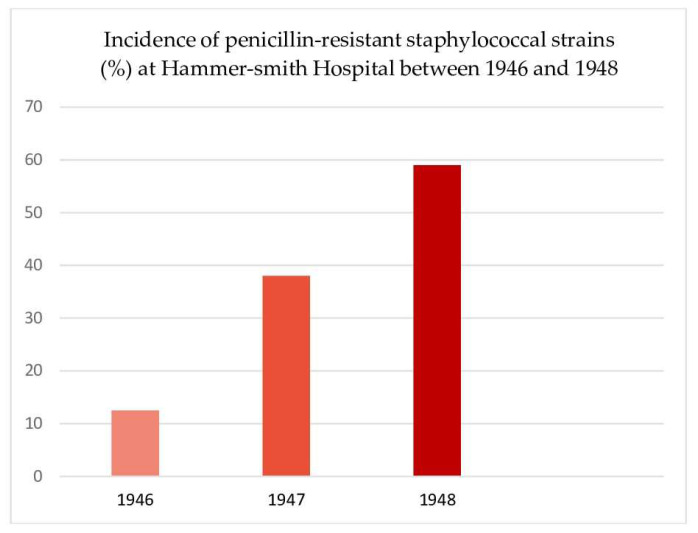
Incidence of penicillin-resistant staphylococcal strains in samples from patients treated at Hammersmith Hospital between 1946 and 1948 (Source: Mary Barber, *Staphylococcal infection due to Penicillin-resistant strains*).

On analysing the frequency of penicillin-resistant staphylococcal strains in samples from patients treated at Hammersmith Hospital, she found very few resistant strains before 1944, while the incidence of such strains increased from 12.5% to 38% between April 1946 and June 1947 [49]. Moreover, in 1948, Barber reported that the incidence had risen to 59% (Figure 4 and Figure 5).

Based on this data, Barber stated that the rise in the incidence of penicillin-resistant staphylococcus strains was occurring so rapidly as to be a cause for concern. She also considered it particularly serious and alarming that the increase appeared to be linked to the spread of a minority of resistant strains within hospitals. Barber was able to demonstrate this resistance in a hospital setting when, the following year, she began working at St. Thomas Hospital. There, Barber discovered that nursing staff contributed significantly to cross-infections in hospitals and that, once they began working within the wards, they quickly became nasal carriers of penicillin-resistant bacteria.

Mary Barber was so impressed by her findings that she soon became a leading figure in campaigns to limit the use of antibiotics. In 1948, Barber wrote: “Penicillin has been discussed so much in both lay and medical circles that the subject seems hackneyed. […] The drug received such acclaim in the early forties that it came to be regarded by many in the nature of a charm, the mere sight of which was sufficient to make all bacteria tremble. Nothing, of course, could be further from the truth and the present widespread and often indiscriminate use of penicillin, particularly as a preventive measure is seriously menacing its future reputation” [49].

According to bacteriologist Mary Barber, it is highly likely that the massive use of penicillin during the Second World War may also have contributed to the development and spread of these first forms of resistance.

In 1958, Barber became a professor of clinical bacteriology at the British Postgraduate Medical School and Hammersmith Hospital. There, she began testing the effectiveness of various strategies to limit the spread of antibiotic resistance. The preventive policies she implemented proved extremely effective in controlling the rise in drug-resistant staphylococcal infections in the hospital.

In the 1950s and 1960s, Barber gave numerous lectures on the topic of antibiotic resistance and published many articles on the subject. In particular, her attention was focused on the spread of resistant staphylococcal bacteria, which she believed had become more virulent due to the widespread use of antibiotics and growing negligence in hospitals regarding the implementation of antiseptic measures. In 1959, she wrote: “the control of antibiotic-resistant staphylococcal infection is an urgent problem and is not simple. It requires the active co-operation of doctors, nurses, physiotherapists, porters and domestics. Scrupulous asepsis and barrier-nursing mean extra work for all concerned. It is readily obtained in a ward where a severe outbreak of infection has recently occurred, but at other times, in the words of Sir Alexander Ogston, ‘human nature forgets unseen foes’” [50].

Still in 1959, the WHO held a meeting dedicated specifically to antibiotic resistance, which was attended by Selman Waksman (1888–1973) and Maxwell Finland (1902–1987) from the United States and Lawrence Paul Garrod (1895–1979) from Great Britain, among others [51].

However, this meeting did not yield great results, as no shared definition of the term “resistance” was found and the World Health Organization itself did not undertake to coordinate the international surveillance or the appropriate use of antibiotics, partly perhaps owing to an excessive trust in the abilities of researchers and the pharmaceutical industry. This trust, however, began to wane in the early 1960s, when it was realized in Japan that bacterial resistance could be spread not only vertically to descendant bacteria, but also horizontally between strains and even species, via mobile genetic elements that would later become known as plasmids.

“Shortly after World War II, (in Japan) a high incidence of sulfonamide-resistant *Shigella* appeared, and, since 1957, *Shigella* strains with multiple drug resistance have been isolated with increasing frequency each year” [52].

In the mid-1960s, following the emergence of so-called “Superbugs”, i.e., bacteria that were resistant to various classes of antibiotics, warnings were issued that “unless drastic measures are taken very soon, physicians may find themselves back in the pre-antibiotic Middle Ages in the treatment of infectious diseases” [53].

## 6. Ancient Antimicrobial Resistance

Just recently, a bacterium embedded in ice dating back 5000 years was discovered in the Scărișoara Ice Cave in Romania. From a scientific point of view, this extraordinary discovery is even more remarkable in that the bacterium is resistant to as many as 10 of the most commonly used modern antibiotics. Research on this bacterium has been conducted by a group of researchers coordinated by the Bucharest Institute of Biology of the Romanian Academy. The results, which have been published in the journal *Frontiers in Microbiology* [54], provide valuable insights into the evolution of antibiotic resistance.

This recent extraordinary discovery could also contribute to indicating new ways to combat antibiotic resistance, as this bacterium has been seen to produce enzymes capable of preventing the growth of other dangerous resistant “superbugs”.

The bacterium in question is a strain of *Psychrobacter* (specifically *Psychrobacter SC65A.3*), a cold-adapted (psychrophilic) bacterium that was extracted from samples of ancient ice containing microorganisms capable of growing at extremely low temperatures. During laboratory analyses, researchers coordinated by Cristina Purcarea discovered that this bacterium was resistant to 10 antibiotics across 8 classes (including third-generation cephalosporins, fluoroquinolones, aminoglycosides, and rifampicin), and that it contained more than 100 genes linked to resistance, as well as approximately 600 genes whose functions are still unknown.

The gradual melting of the Earth’s ice could release these microorganisms into the environment, potentially facilitating the dissemination of resistance genes among contemporary bacterial populations. This phenomenon could further aggravate the global challenge that has faced us for years: the fight against antibiotic resistance.

On the other hand, the authors also identified 11 genes that contain the instructions for producing enzymes and other substances that can potentially kill or block the growth of other bacteria, viruses and fungi, suggesting that ancient microbial reservoirs may also represent a valuable source of new antimicrobial agents.

## 7. Conclusions

Antimicrobial resistance constitutes a critical threat to public health, demanding urgent and effective policy action to limit its consequences.

The historical evidence discussed in this review highlights that the ability of microorganisms to withstand antimicrobial agents predates the antibiotic era.

The cases examined show how, even in the earliest phases of antimicrobial chemotherapy, microorganisms were capable of developing mechanisms to survive drug exposure.

The extent and impact of this phenomenon have been exacerbated by the widespread and often inappropriate use of antimicrobial agents in clinical, agricultural, and environmental settings.

Significant efforts are needed to strengthen strategies to combat AMR, including improvements in microbiological diagnostics, particularly through the implementation of rapid and molecular tests, to enable timely and targeted therapies and reduce reliance on inappropriate empirical treatments. At the same time, the development of new therapeutic strategies, such as innovative molecules, optimized drug combinations, and alternative approaches, remains essential.

## Data Availability

The original contributions presented in this study are included in the article.
